# How Did the Archaellum Get Its Rotation?

**DOI:** 10.3389/fmicb.2021.803720

**Published:** 2022-04-26

**Authors:** Davi Ortega, Morgan Beeby

**Affiliations:** ^1^Koan Labs, São Paulo, Brazil; ^2^Department of Life Sciences, Imperial College London, London, United Kingdom

**Keywords:** archaellum/archaea/archaellum motor complex, propulsive nanomachines, archaeal flagella, genomics, biophysics, *in situ* structural biology, molecular evolution

## Abstract

How new functions evolve fascinates many evolutionary biologists. Particularly captivating is the evolution of rotation in molecular machines, as it evokes familiar machines that we have made ourselves. The archaellum, an archaeal analog of the bacterial flagellum, is one of the simplest rotary motors. It features a long helical propeller attached to a cell envelope-embedded rotary motor. Satisfyingly, the archaellum is one of many members of the large type IV filament superfamily, which includes pili, secretion systems, and adhesins, relationships that promise clues as to how the rotating archaellum evolved from a non-rotary ancestor. Nevertheless, determining exactly how the archaellum got its rotation remains frustratingly elusive. Here we review what is known about how the archaellum got its rotation, what clues exist, and what more is needed to address this question.

## How Does Rotary Motion Evolve in Molecular Machines?

How do molecular machines evolve new roles? Macroscopic structures such as wings or eyes have evolved from simpler limbs or photoreceptors. In many cases, evolution of new functions in such macroscopic structures involved co-option of a pre-existing feature for the new role. Gould and Vrba coined the phrase *exaptation* to define this process, distinguishing it from *adaptation*, in which a pre-existing feature evolves to become more beneficial in its current role. Lightweight feathered wings, for example, existed in nascent forms before exaptation for flight (Gould and Vrba, 1982).

How such processes operate at the molecular scale remains less well understood. Nevertheless, it appears that at the molecular scale new functions also result from exaptation of existing underlying mechanisms instead of radical genesis of fully formed new machines. For example, rotary nanomachines used by cells for propulsion demonstrate exaptation of rotary mechanisms (Beeby et al., 2020). The best known such machine is the bacterial flagellum, which rotates a multi-micron filament as a rotary helical propeller to generate thrust. Bacterial flagella are believed to have exapted the intrinsic rotary mechanism of a family of rotary ion channels (the ExbBD/TolQR family, discussed elsewhere in this special issue), to drive rotation of a large axial ring, cogwheel-style, that in turn rotates an extracellular helical propeller (Chang et al., 2020; Deme et al., 2020; Santiveri et al., 2020). Better understanding of how this exaptation occurred, however, is frustrated by the absence of contemporary flagellar relatives that diverged prior to the evolution of rotation, leaving the evolutionary path to rotation speculative (Abby and Rocha, 2012; Beeby et al., 2020).

If the emergence of novelty in molecular machines involves exaptation, evolution of rotary motors suggests that rotary function should be a nascent function of ancestral protein components. Four decades ago, Roger Hendrix suggested that rotation may be inherent to symmetry mismatched protein subcomplexes (Hendrix, 1978): the phase shifts in binding interactions around the interaction surface of two symmetry-mismatched cyclic subcomplexes will reduce the activation barrier to the small rotations required to achieve an identical–albeit rotated–set of interactions. In other words, unless active measures are taken to prevent rotation, rotation may be inevitable in such cases. Hendrix based his thesis on symmetry mismatch in phages, and symmetry mismatch was subsequently implicated as important for function of the bacterial flagellum (Thomas et al., 1999), ATP synthase (Pogoryelov et al., 2005), Clp proteases (Ripstein et al., 2020), kinetochore movement along microtubules (Westermann et al., 2006), and DNA translocases (Liu et al., 2015).

Curiously, rotation is not as frequently seen as we anticipated. Since Hendrix published his work, the phage portal protein has been shown not to rotate (Hugel et al., 2007), the protease ClpAP has been shown to function without rotation (Kim et al., 2020), and diverse symmetry-mismatched secretion systems have been shown to have architectures that preclude rotation (Umrekar et al., 2021a). Together, this suggests that strategies such as the sophisticated interlocking of different symmetries in phages (Fang et al., 2020) may have evolved to preclude the potential side-effect of inter-subcomplex rotation (Liu et al., 2014). If this assumption is the case, how might a protein machine overcome a selectively detrimental state to exapt an intrinsic rotary mechanism?

“How” is a nebulous word, and we use it to capture diverse questions: has exaptation played a role in the evolution of rotary protein machines? How many mutations are required to switch from a non-rotary interface to a rotary interface? What selective pressures exist to retain a protein complex in a non-rotary or rotary state? What structural features implement the retention of one or the other state (for example, symmetry adaptor domains, proteins that mediate stoichiometry matching, or promiscuous inter-subunit binding interfaces)? Do (or must) selectively metastable “gateway” evolutionary intermediates exist that facilitate the evolutionary pathway from non-rotary to rotary function? Such questions remain broadly unanswered, in part because we lack a model system in which to ask them.

## The Archaellum: An Excellent Case Study of the Evolution of a Rotary Motor

**FIGURE 1:**
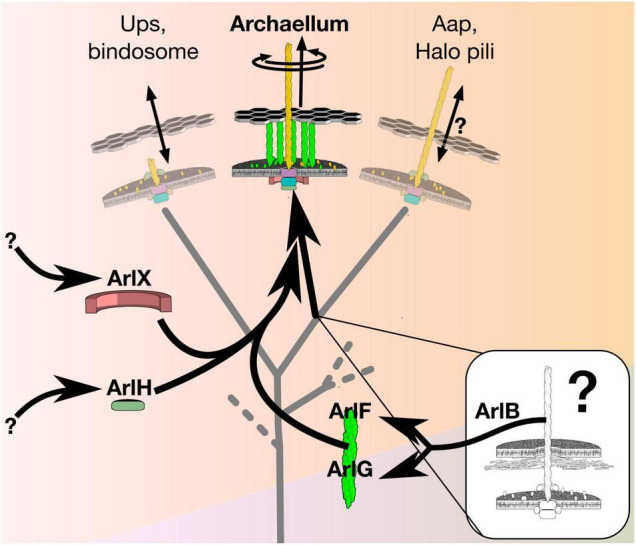
The archaellum is a rotary motor that evolved from a non-rotary ancestor. The archaellum descends from a clade of the type IV filament superfamily that produced adhesive pili. Evolution of rotary motion included paralogous duplication of its filament protein, ArlB, to form the putative stator ArlFG, recruitment of ArlH from an unknown source as putative timer protein to switch from assembly to rotation, and recruitment of ArlX (or, in some species, ArlCDE) from an unknown source as a putative stator scaffold. The details of these recruitments, and how they affected the function of intermediate states, however, remains unclear.

A possible model system to study emergence of rotation in molecular machines is the “archaellum” (previously known as the “archaeal flagellum”), whose diverse non-rotary contemporary relatives provide clues to its evolution (Albers and Jarrell, 2018). Archaella are members of the type IV filament (TFF) superfamily, a family of otherwise non-rotary machines that includes type II secretion systems, type IVa pili, type IVb pili, and various adhesin pili (Berry and Pelicic, 2015; Beeby, 2019; Denise et al., 2019), but feature additional proteins apparently associated with torque generation (Figure 1). Like bacterial flagella, archaella spin a filament to form a helical propeller that generates thrust for cell propulsion.

Although the archaellum is less well-known than other rotary motors, its simplicity makes it an excellent system for studying the evolution of rotary function. The simplest archaellum, from *Sulfolobus acidocaldarius*, features just seven proteins. A multimicron-long extracellular filament of ArlB assembles on a platform composed of transmembrane ArlJ and cytoplasmic ArlI and ArlH; paralogs ArlF and ArlG likely span the pseudo-periplasm, while ArlX may mediate complex formation between ArlHIJ to ArlFG. (Note that archaellar proteins have recently been renamed “Arl” from “Fla/Flg” to prevent confusion with bacterial flagellar proteins Pohlschroder et al., 2018).

The ArlHIJ complex lies at the heart of the archaellum. Transmembrane ArlJ features two conserved cytoplasmic domains (Ghosh and Albers, 2011), dimerizes, and likely interacts with the cytoplasmic ATPase, ArlI, a cyclic hexamer that is the sole energy source of the complex. Curiously, ArlI powers both archaellum assembly and rotation, and it is a conserved member of the same ATPase family used across other members of the TFF family. These ATPases drive filament assembly in all TFF superfamily members; some family members also feature paralogs that can retract the filament; some family members have multiple paralogs capable of retraction with different forces (Chlebek et al., 2019; Talà et al., 2019). Beneath ArlI lies ArlH, which features ATP-binding motifs but has not been shown to be an ATPase (Hanson and Whiteheart, 2005; Chaudhury et al., 2016). ArlH has a RecA/RAD51-like fold related to the circadian clock protein KaiC (Chaudhury et al., 2016; Meshcheryakov and Wolf, 2016), suggesting a timer function implicated in triggering a switch in ArlI between its two roles in assembly and rotation.

ArlB forms the archaellar filament, which coils to form a helical propeller when rotated by the archaellar motor (Poweleit et al., 2016; Daum et al., 2017; Meshcheryakov et al., 2019). This filament is assembled atop ArlJ using energy from ArlI’s ATP hydrolysis activity. The ArlB filament is a compact helix assembled around a bundle of hydrophobic N-terminal α-helices. The head and N-terminal helix interact with six or eight neighboring protomers to assemble a strong and relatively rigid filament.

As with all rotary motors, productive rotation must depend upon a static stator component that the rotor component can rotate relative to. In the case of the archaellum, ArlF and ArlG have been implicated to form this stator (Banerjee et al., 2015). Both localize to the archaeal pseudo-periplasm; ArlG forms a helical filament that is probably capped by S-layer-anchored ArlF; deletion of either gene or abolition of S-layer binding abolishes rotation (Tsai et al., 2020).

The final component of the archaellum is an enigmatic cytoplasmic protein complex. In the model crenarchaeon *S. acidocaldarius*, ArlX, a protein with a transmembrane helix, forms 30 nm-wide cyclic oligomers (Banerjee et al., 2012, 2013). Other species instead feature a complex of ArlCDE. These lack predicted transmembrane helices but feature sequence motifs that evoke ArlX, although whether ArlCDE and ArlX are homologs remains to be seen. For brevity, for the remainder of this text we use “ArlX” as shorthand to denote both ArlX and ArlCDE. Little more is known about the role or location of ArlX.

### What Is Known About the Mechanism of Archaellar Rotation?

Rotary motors rotate a rotor component against a stator component. In many, including human-made motors, the ATP synthase, and the bacterial flagellar motor, the energized component is integral to the stator, harnessing an energy source to drive rotation of the passive rotor. It remains unclear whether the archaellar energized component is part of the stator or rotor, however, and it is unclear for many components which rotate and which remain static–except for the S-layer-anchored ArlFG complex and rotating ArlB filament (Shahapure et al., 2014; Kinosita et al., 2016). While it follows that ArlJ, the assembly platform for ArlB, is contiguous with the archaellar filament to prevent the ArlB filament from detaching and diffusing away, *in situ* structures of related type IVa pili suggested that the ArlJ homolog could rotate against the pilus (Chang et al., 2016). The ArlJ:ArlI or ArlI:ArlH interfaces are candidates for intersubunit rotations because ArlI homologs undergo 60° rotations of conformational states, suggesting that this might be converted into physical rotation by pushing against ArlJ or ArlH. Indeed, conflicting evidence for the oligomeric state of ArlH *in situ* highlights the possibility of symmetry mismatch-mediated rotation between ArlI, ArlH, ArlJ, or ArlX. Nevertheless, ArlI, ArlH, and ArlX interact, suggesting they form a contiguous complex (Chaudhury et al., 2016), and ArlX is destabilized in the absence of ArlJ, suggesting that it is a structural component associated with the core of the motor. These insights hint that the rotor:stator interface may instead lie between ArlX and ArlFG; conformational changes in ArlI resulting from ATP hydrolysis could be communicated via ArlX to exert force against an interface with ArlFG, resulting in ArlFG being the stator and ArlX, ArlH, ArlI, ArlJ, and ArlB being the rotor. Nevertheless, ArlX would also provide an obvious structure to anchor a ring of ArlFG stator units. Understanding the location of this elusive interface, and identifying the mechanism of torque generation, is crucial to understanding where the archaellum got its rotation.

Direct observations of archaellum rotation provide some clues to the underlying mechanism (Alam and Oesterhelt, 1984; Shahapure et al., 2014). Archaella rotate both clockwise and counterclockwise but do not extend or retract during rotation. The torque produced by the archaellar motor is of the magnitude expected from ATP hydrolysis by a single hexameric ATPase (Iwata et al., 2019): the estimated torque required to rotate the archaellum one full turn would require hydrolysis of 12 ATP molecules, suggesting that two of the hexameric ArlI ATPase subunits are active at any one time, which coincides with the dimeric nature of ArlJ (Iwata et al., 2019). This, in turn, corresponds to detection of 60° steps in archaellar rotation in both directions (Kinosita et al., 2016), although a confounding observation is that the archaellum also rotates in 36° steps in both directions.

### The Current Model for How the Archaellum Got Its Rotation

Understanding the evolutionary steps required to bridge the gap between a non-rotary ancestor to a rotary archaellum may be within our reach. Two phylogenetic studies of the TFF family provide first clues into how the archaellum evolved (Makarova et al., 2016; Denise et al., 2019). The more recent study assumed that archaellar components are organized in gene clusters in order to annotate TFF family members across bacteria and archaea, revealing that archaella diverged from a non-retracting pilus clade of the TFF family that includes Aap pili, halo pili, UV-inducible pili and bindosomes (Denise et al., 2019), suggesting the non-rotary ancestor of the archaellum assembled such a pilus. Archaella differ from this putative ancestor in three ways: first, their ATPase can generate torque; second, their ATPase can switch from assembly to torque generation; and third, they feature a stator complex against which to exert this torque. These differences involved gain of ArlF, ArlG, ArlH, and ArlX.

Naively, the biggest step would have been the first: to evolve an ATPase that exerts torque. It may be, however, that the intrinsic rotation of conformational states in the hexameric AAA + ATPase family was exapted for rotation of the rotor. It has even been suggested that all TFF ATPases rotate while extending the pilus (Campos et al., 2013) due to a symmetry mismatch between the hexameric ATPase and the helical pilus, suggesting that TFF ATPases rotate within the cytoplasm while the pilus extends. This, however, may be a red herring, as it holds that assembly is integral to rotation; the archaellum, meanwhile, can still rotate after assembly has halted.

What is required, then, is the second step: evolution of a stator complex to anchor to the cell superstructure to exapt the rotation of ArlI to rotate the pilus. Curiously, the probable stator proteins ArlF and ArlG are paralogs of the filament protein ArlB (Banerjee et al., 2015; Tsai et al., 2020). ArlF and ArlG share a fold and binding interfaces with ArlB, although ArlG forms an open helix, unlike the more rigid closed ArlB filament (Umrekar et al., 2021b). Although superficially dissimilar, closer inspection reveals that ArlG protomers still retain a subset, but not all, of the inter-protomer interactions of ArlB protomers, resulting in loss of lateral interactions and fewer ArlG protomers incorporating into the helix. Such an open helix of the ArlG filament will be more flexible than the tightly interconnected ArlB filament and could facilitate elastic storage of the energy burst from ATP hydrolysis. ArlX may couple the ATPase to the stator complexes.

The remaining step, functional switching of the ATPase from assembly to rotation, may not have been essential, as a rotating pilus that continues to extend, while wasteful, would still generate thrust. Switching from assembly to rotation may have initially been achieved by chance, and later enhanced by adding a dedicated ArlH timer. Indeed, because measuring the length of the archaellar filament is difficult, a timer may have been easier to evolve than a ruler.

## What Is Needed to Understand How the Archaellum Got Its Rotation?

### More Information on Diversity That Might Reveal the Evolutionary Path to Rotation

Understanding how the archaellum evolved to rotate will benefit from the most comprehensive possible view of (meta)genomic diversity to understand contemporary variants and to ascertain whether any proto-archaellum “missing links” exist. Earlier studies lacked the current wealth of (meta)genomic data available, or assumed that all archaellum components cluster together in a genome (Makarova et al., 2016; Denise et al., 2019). Their sequence models can serve as a foundation for future focused studies across the breadth of genomic diversity (Parks et al., 2021) and can be updated to generate more sensitive models. False positives can be removed by iteratively removing proteins established to function elsewhere. Whether an identified TFF is an archaellum can also be suggested based on the presence of a class F1 chemosensory system (Wuichet and Zhulin, 2010), which appears to signal exclusively to archaella in the Archaea (Briegel et al., 2015). Most chemosensory proteins are easy to detect and unique to the chemosensory pathway.

A phylogeny derived from concatenated alignments of ubiquitous components of only archaella and close relatives will allow us to understand sequence changes during evolution and, ideally, “missing links” that lack one of the proteins exclusive to archaella. Should such a missing link be discovered, it will be valuable in illuminating the evolutionary steps taken to form the first fully fledged contemporary archaellum. Such missing links, however, are notoriously elusive, and they have still not been discovered for analogous, intensively studied machines like the bacterial flagellum (Beeby et al., 2020). Absence of these intermediary steps is similar to gaps in fossil records (Gould and Eldredge, 1993) and is suggestive of punctuated evolution, in which the first rotary archaellum rapidly (almost instantaneously on the evolutionary timescale) adapted to form an archaellum that already resembled that of contemporary *S. acidocaldarius*. Anecdotal support of this may come from the (admittedly problematic) use of core functionally invariant components as molecular timekeepers. For example, selective pressure on the prepilin peptidase, ArlK (Bardy and Jarrell, 2002), may not have changed in the transition to becoming an archaellum from a pilus, making ArlK a potential chronometer against which to assess rapid punctuated evolution of other components.

The diversity in bacterial flagella was unanticipated until they were imaged (Chen et al., 2011). Similarly, archaella may also have such unanticipated diversity. Comparative genomics may highlight variants of archaella and predict novel components. Consistent operon co-occurrence of two genes is a strong indicator of functional interaction of their products (Overbeek et al., 1999) and may suggest novel archaellum components. Similarly, curated phylogenetic profiles of protein families can highlight consistent cross-genome co-occurrences of protein families that are candidates for novel archaellum components (Pellegrini et al., 1999).

### More Information on the Mechanism of Rotation

To understand how archaellar rotation evolved, it will be crucial to understand the mechanism of rotation. Obtaining atomic models of the archaellum in different conformational states is now conceivable given recent advances of *ex situ* and *in situ* cryoEM and structure prediction (Baek et al., 2021; Jumper et al., 2021). The best candidate for such structural work is likely to involve electron cryotomography with subtomogram averaging of the archaellum from *Pyrococcus furiosus*, which has proved to be the most tractable system for *in situ* archaellar imaging (Daum et al., 2017). More data collected using contemporary hardware will be required to push to the ∼8 Å-resolutions required to position α-helices.

By combining such structural snapshots of the machinery in action with biophysical studies and molecular dynamic simulations, it may be possible to parameterize a predictive molecular model of torque generation. Biophysical measurements will require high temporal and spatial resolution of torque and speed to identify steps and torques, mapped to different motor conformations using correlated structural and cellular techniques. Perturbations to mechanical output in mutants will provide more information about the roles of subcomponents. For example, the recent determination of the structure of the ArlG filament suggests that it acts as a stator and as an elastic storage device for the energy released during ATP hydrolysis (Umrekar et al., 2021b). Would a more rigid ArlG filament change the mechanical output of the motor by making steps more abrupt?

Understanding these results will ultimately also require us to understand which components rotate and which remain static, a goal that will require sophisticated imaging of live cells to detect rotation of different archaellar labeled subcomponents. Combining this with high resolution subtomogram averages of functional archaella *in situ* will enable mapping of conformational states to show what rotates and what does not.

### More Information on the Origin of Rotation: Was It an Exaptation of Intrinsic Type IV Filament Rotation?

Is rotation intrinsic to TFFs, and was it exapted for rotation in archaella? Success in understanding which proteins rotate and which remain static may be extended to components of other TFF members to test whether proteins that rotate in archaella also rotate in other family members. Alternatively, a mutant could be created that binds or crosslinks (using, e.g., cysteines or unnatural amino acids) components to the cell body to create a surrogate for a stator complex to see if this results in pilus rotation.

Ultimately, the acid test of our theories will be whether we can recapitulate the evolutionary events we infer. First steps will be to “break” the archaellum in targeted components so that it no longer rotates and to identify suppressor mutations that restore motility. What is required for this? More extensive efforts may generate plausible ancestral states using ancestral sequence-reconstruction techniques to timepoints along the evolution of the contemporary archaellum and attempt to recapitulate the subsequent steps using strong lab-based selective pressure. Yet more ambitious would be to attempt to evolve an archaellum analog from a contemporary TFF superfamily member.

## Concluding Remarks

The time is ripe for us to make substantial advances in understanding how rotation emerged in a molecular machine. The archaellum is a rotary motor that is uniquely simple (with many fewer components than all other rotary motors except the ExbBD family), easy to assay (with a long filament whose rotation is easier to monitor than complexes such as ExbBD), and phylogenetically well-understood (with many non-rotary cousins), making it an ideal case study of the emergence of rotation. Recent and continued advances in genomics, biophysics, and structural biology suggest that the archaellum is poised to become the first rotary machine whose evolution we understand.

## Author Contributions

Both authors conceived, wrote, and edited the manuscript.

## Conflict of Interest

The authors declare that the research was conducted in the absence of any commercial or financial relationships that could be construed as a potential conflict of interest.

## Publisher’s Note

All claims expressed in this article are solely those of the authors and do not necessarily represent those of their affiliated organizations, or those of the publisher, the editors and the reviewers. Any product that may be evaluated in this article, or claim that may be made by its manufacturer, is not guaranteed or endorsed by the publisher.
